# Obscure Pains in and about the Jaws

**Published:** 1892-01

**Authors:** J. E. Garretson

**Affiliations:** Philadelphia


### OBSCURE PAINS IN AND ABOUT THE JAWS.[1]

BY J. E. GARRETSON, M.D., D.D.S., PHILADELPHIA.

Before reseating myself, gentlemen, you will undeniably be impressed that if your guest is offering anything worthy of acceptance on the part of the members of this Society, his own occasion is great for something to carry away. It is something, however, for one to know that he knows what he knows and to know that he does not know what he does not know. From the stand-point of this Confucian aphorism as designative of reliable knowledge, so far as it goes, I venture on a consideration of obscure pains in and about the jaws.

---

[1] Read before the New York Odontological Society, October, 1891.

By obscure pains is meant pains the causes of which are more or less evident or not evident at all.

*First.* As to pain. What is pain? Abstractly it is to be assumed that all here will agree that it is altered sensation.

*Second.* If sensation is altered, the alteration is to be accepted as lying with a cause.

*Third.* Cause lying back of pain in every instance, the term obscure pain resolves itself into one of ignorance of cause.

*Fourth.* Obscurity being one with ignorance, the common term shows itself to be relative,—that is to say, the obscure to one is the open to some other one. There is another way of putting this in connection with the present occasion,—viz., it is not impossible, by reason of work done by members of this Society, that the term "obscure," as applied to pains in and about the jaws, from the stand-point of an individual's experience and knowledge, may, by reason of a putting together of heads, find to-day a complete revelation.

By reason of contracted outlook we lack in ability to read causes as these relate with variety. So we come to perceive that professional knowledge—and this is very encouraging to the young men, as well as to we older ones—is a matter of study and experience: finding out what others know; keeping up with the literature of our profession; taking advantage of opportunities to observe; learning something to-day which we did not know yesterday, and hoping to get something new to-morrow; and so if some case comes to us to-day that we cannot treat properly from lack of knowledge, to-morrow we may trust to find ourselves improved in that particular direction.

Pain existed in the time of Galen as in the present time. You recollect the saying attributed to him that "the cause of toothache is known alone to God." The Chinese are credited with the discovery of cause of this particular ache as lying with a worm, and deemed him ignorant who required other proof than was to be found in splitting a complaining grinder. Query, To what does the extreme modern bacteriologist attribute pain? Extremes meet.

Let our subject here find opening. Appreciation of the causes of pain finds understanding in diagnosis, as this lies with the processes of exclusion,—that is, putting it in other words, finding out what a thing is by first finding out what it is not.

Let me assume to say what I have to say as to searches made after diagnoses, as illustrated in procedure, which has become by reason of long usage a kind of second nature to me.

A patient entering my clinic or office is assumed as coming by reason of not being in a state of ease; not being in a state of ease, the condition is its converse,—viz., dis-ease. As students we long ago resolved this term.

The prefix "dis" expresses a converse happily and fully. If a man be not honest, we say he is dishonest; one not being in a state of comfort, he has been discomforted; not being able, he has been disabled. The ability to say what a "dis" is depends on education. Ask a laboring man on the street as to the cause of toothache, and he will not give you a very good answer. If you ask a young student, he may not give you a very good answer either. Men find themselves diagnosticians according to understanding, education, and acquirements. We are diagnosticians proportionately with employed opportunities. Diagnosis by exclusion lies with recognition of the existence of things pertaining to a circle around which the art of diagnosis is to move. Discrimination is with differentiation. A diagnostician is to know, for example, that a particular circle contains or embraces factors; let us assume a circle having twenty factors. The virtue of his art consists in saying correctly as to a particular factor out of a possible twenty related with this circle which may be at fault. It is the extreme of his art to be able to demonstrate that a *dis* lies with a twentieth undiscoverable cause in showing that it is not any other one or more of nineteen other causes or *dis*es, to the discrimination of which his knowledge is equal.

To make illustration. In the museum of our college at Philadelphia is the skull of a man who suffered untold agony from neuralgia affecting all the branches of the right trifacial nerve. No diagnosis was reached, nor was any one of a multitude of agents administered for his relief of the slightest service. Post-mortem examination revealed a spicula of bone growing from the border of the oval foramen and piercing the nerve: This case may well be set down as the twentieth cause of a circle to be considered,—a circle of jaw pains the first factor of which we shall assume to be a tooth decayed to an extent that exposes its pulp. The latter is simplicity itself; the former is identical with obscurity. To be able to know or to diagnosticate any pain in or about the jaws is one with knowing about all the possible factors. Who of us are so wise?

There are cases of obscure pain in the jaw regarding the origin of which we are entirely at a loss. We ask ourselves vainly as to the "dis." If the experience of the members of this Society is akin with mine, they often find themselves compelled to wait a long while before finding out so simple a matter as that a supernumerary tooth is working itself down through a bone, and is worrying it.

Just before coming here, I had a peculiar case in a young gentleman sent from a distant State, who was suffering from a trouble situated about a tuberosity of the upper jaw. As I happened to cure him, I may explain that it was done by reason of a greater familiarity with a circle of possible causes than was possessed by a physician at whose hand he had been treated. I found on examination nothing particular out of the way in his jaw, except an undue broadening of the part complained of. It looked like an ordinary hypertrophy, not like a congestion. I made a cut through the swelling, finding what I had never met with before,—a supernumerary wisdom tooth.

Another case may be offered as being suggestive. Dr. Roberts, who, by the way, has died lately, had a conversation with another dentist, about a patient who was inferred to have cancer of the upper jaw. It was certainly a very ugly-looking jaw. It was in a softened, carious, broken-down condition, and the supposition seemed reasonable that the lesion was that of sarcoma. Examination impressed me that it was not unlike ordinary cases of sarcoma, as these are met with in clinical practice. After two or three visits I proceeded to scrape away the soft and offensive mass, and found situated in the nasal process of the maxillary bone a long eyetooth. I took the tooth out, and the man got well in a few weeks. Not only the man in the street, but also some of our young friends, would have said, upon seeing such a jaw, "This is cancer." That is certainly what I thought on first looking at it.

I had a case recently, where a superior maxilla was seen to be very much enlarged externally; but when I looked into the mouth, I found the jaw edentulous, and yet in one of this man's antra I exposed five teeth.

Let us review or recall diagnostic signs as lying with pain itself.

We are to remember that, by reason of causes or conditions, pain varies from simple departure from ease to unbearable agony, and this not at all as significant of danger or the absence of it, but as expressions of location; example of the former is to be made by reference to pneumonitis, or, better, to Pyer's patches in typhoid; of the latter to pulpitis. All are familiar with the stitch in the side of pleurisy. To hear this last complaint from a person leads at once to the diagnosis of pleuritis. Acute and throbbing pains in the region of cellular tissue points with certainty to a developing boil or carbuncle. Obtuse and heavy pain located about the liver or lung points to inflammation of these organs.

A bone inflammation is expressed in a sense of gnawing; a skin inflammation is located, even under one's unremoved clothing, by an itching combined with burning that is indicative of it; the conjunction of itching about the glans penis and stone in the bladder is familiar; coxalgia, as pointed out by pains at the knee; neuralgia, as differentiated from inflammatory pain by its sharp and lacerating and shock-like expression; throbbing, as denoting approach to suppuration, etc.

With these few but sufficient general premises as foundational, let us pass to the details of our subject as lying with the process of exclusion.

The first and most common cause of jaw pain is odontalgia; significant of,—

1. Exposure of tooth-pulp.
2. Peridentitis.
3. Sensitive dentine.
4. Confinement of gas or pus in a closed pulp-cavity.
5. The presence of granules of osteo-dentine in a pulp.
6. The recession and absorption of gums and alveolus.

These are familiar, every-day experiences, too common to need else than attention being called to them in a preliminary way. To these are to be added the pains of the dentitional period, etc.

Following these I would place syphilis of the tertiary form; undeveloped teeth: first, wisdom-teeth of lower jaws; second, supernumerary teeth; third, anomalous or unique growths.

Next, rachitic conditions in children: the discomfort lying here in a loose relation of the teeth with their sockets. Diagnosis is plain, relating as it does with a prevailing cachectic state.

I would place next, drawing on my own experience, pains in the mental region of the lower jaw that strike a patient suddenly, increase gradually, being soon seconded by a puffy inflammatory enlargement of the whole chin region, indicative of a free effusion of a plastic lymph, and which has its termination invariably in the loss of the teeth of the region by reason of exfoliation in the shape of sequestra of the alveolar process.

Next is to be placed a dull, heavy, demoralizing kind of pain, situated in the region of a cheek, where cause lies with closure of an artificial opening of an antrum, in which inflammatory change has destroyed the ciliated expression of the mucous lining of the cavity, and where, consequently, the secretion lodges and remains upon the floor of the cavity.

I have had patients come to me from the distance of a thousand miles to consult, where the whole fault, if I may be pardoned for the suggestion, lay in the attempt of a dentist or physician to close a break in the antrum. I do not allude to those slight breaks that occur in extracting teeth, for such breaks close themselves. The condition referred to is where there has been a degenerative inflammation that has removed a portion of the floor by sequestration. Physiological changes in the mucous membrane have here occurred, which make closure of the break one with retention of the secretion. In my own practice, where an antrum has remained open some time, and where there has been a general inflammation about the part, I always advise keeping the break open.

Next, erysipelas. This specific inflammation, when appearing upon the face, expresses itself primarily as of deep origin. A person will complain of pain about the head and jaw, situated principally over the malar bone. The part is sore on pressure. You look into the mouth of the person, but see nothing. You wait a few hours, and now find a developing tension. Here is plainly shown a perversion of the circulation, indicative of inflammatory change. A little later the blush expressive of erysipelas is seen. I would like just here to throw out a suggestion to the effect that I have never known a case of local erysipelas that would not yield to a direct application of iron, quinine, and cinchona. A wide-thinking man may accept this specific, yet will not be deterred asking as to probable constitutional conditions. The prescription is as follows:

℞ Tinct. ferri chlor., ℥i ;

Quiniæ sulphatis, ʒi ;

Tinct. cinchonæ, ʒii. M.

The affected part is to be painted and repainted until the red blush disappears.

The means suggested is not an agreeable application ; it blackens the part. I have found from hospital experience that the ordinary benzoinated oxide of zinc ointment is a salve that may at times happily and efficiently replace it.

Next, malarial pain. Malarial pain finds expression, malaria being the "dis," in a lack of comfort about the teeth that is not easily described. The patient complains of an uneasy sensation in the organs. You find them perfect, perhaps, and are led to an inference of malarial relation through practise of the process of exclusion. Taking, say, twenty possible causes, of which malaria is set down as the twentieth, and not finding the trouble related with any one of the nineteen other causes, one alone remains, namely, malaria.

Do we know certainly how many causes there are in the circle of jaw pain? No, we do not. So, sometimes we must feel our way by means of treatment. First, in a case of inferred malarial poisoning, as just referred to, the way is felt to an endorsement of our conclusion by putting a person on antimalarial treatment,—not big doses of quinine, because that is not always good. If any of you have lived in a malarial region, you know that accompanying the malaria there is a sense of depression and a sense of good-for-nothingness,—a patient not being absolutely sick,—certainly not reasonably well. I take the liberty to suggest in such connection a remedy which, when you are feeling your way for a diagnosis, may do some good in way of helping both you and the patient. I know of but few instances in which malaria has not yielded to this prescription,—

℞ Cinchonæ rubra pulv., ℥i;

Serpentariæ Virg., ℥ss. M.

To be put into one and a half pints of water, simmered into one pint, strained when cold, and one pint of Lisbon wine added. The dose is two tablespoonfuls three times a day; best taken just before meals.

Next, the so-called neuralgiæ. To say simply that one has neuralgia is, of course, but to repeat in English words the classic term.

The causes of so-called neuralgic pains are various; these being of local or systemic meaning. The most common seats of these pains are that of the supraorbital region, superficially, and of the line of the anterior portion of the superior longitudinal sinus deeply. The pains of these regions are commonly due to eye-strain. I had yesterday in my office a lady who has been suffering for a long time from such pain. There is such a continuance of discomfort that she has felt that life was not worth living. It was natural for me to look at her teeth. I found nothing the matter with them. Then I began to go around the circle, and, finally, I came to her eyes, discovering that she was myopic in the left eye to the extent of a sixth, and in the right eye to the extent of a fifth. I found something else, and this was perhaps of more importance than the first, —she had an insufficiency of the superior rectus muscle of the right eye, requiring a fifth prism for its correction, and similar insufficiency with the inferior rectus of the left eye. Take these conditions, and I think you will readily recognize how a person could be a continuous sufferer.

Once I had visit me a surgeon of the United States navy who had come from China to his home, under the impression that he had softening of the brain. He told me he had consulted many physicians who came into the Chinese waters without having any relief given him. The pain was continuous. I examined that man's mouth first, and found he had one of the most beautifully filled upper bicuspid teeth that I had ever seen. That was the only thing I could see in the way of a diagnosis. I extracted the tooth. The pain was gone quick as a wink. Think of the relief to that man!

Here, in the last two cases mentioned, are opposite aspects,—the one, muscular insufficiency; the other, an irritation of a branch of the fifth nerve. It does not imply, as to the first, that the pain came from a muscular radicle; it might have come from some distant part. I want only to impress that pain lies in cause, and that we should find ourselves able to expose cause, whatever or wherever it may be.

I could mention from my own experience a number of cases where insufficiency of the recti muscles has proved the cause of pain. Take a person, for instance, who focalizes well. He will take up a paper and read it perfectly. The accommodation is all right; but after reading any time, from ten minutes to two hours, the letters begin to blur and pain comes about the head. Almost always the trouble is found here to lie with muscular insufficiency. If such fault be corrected the pain is cured. Another thought is to be thus expressed: a man has long prided himself on having grand eyesight; take one of our own profession,—he has worked for years untiringly; finally, he operates for only half a day, and says, "I cannot work as I used to. I feel that the power is leaving me." His trouble lies, more than likely, in a presbyopic condition of his refractive apparatus. He needs a plus lens. Take a piece of ordinary window-glass and grind it down to a sixtieth convex, and his power is found to be in its old place. But if our colleague knows nothing about plus glasses, he goes on in a misconception of his condition that proves the destroyer of his usefulness.

Next, reference is to be made to pain located within the lower jaw, or external or internal to it. Inflammatory thickening of the inferior dental nerve always presents, as I incline to accept, a certain easily observable tumidity of the gum-tissues overlying the bone. This being seen of persistent character, the indication is for exposure of the nerve in its canal and its removal; but if the latter stages of secondary or the initial conditions of tertiary syphilis be evident, or if rheumatism be present in other parts of the body, operative procedure will be wisely delayed until fair trial is given of the iodide of potassium.

Just here I have a personal experience to offer that I am sure is of large value. It relates with cases where removal of the inferior maxillary nerve has been practised in the canal, no benefit resulting, but where cure follows neurectomy done at the foramen ovale. Differences as to result exist here, with the fact that it is the mylo-hyoid branch that is involved. Again, full and complete neurectomy of the inferior maxillary nerve, unattended with cure of neuralgia, has satisfied me that the source of pain lies in the incisive branch of an opposite nerve that has passed beyond its ordinary line and is prolonged in its neighbor's canal. Pain confined to the mental region of one side finds explanation most frequently in a lesion existing at the mental foramen.

I think this matter about tumidity must certainly be new to some of you, for I have never seen it referred to in a book. A person comes to you with neuralgia in the lower jaw. You perhaps have had a great many cases of neuralgia of the lower jaw, and have not done much with them; but I think you will find that always where there is a thickening of the nerve-sheath, or where the lesion lies with the nerve-canal, there is always an external exhibit in tumidity. This is my individual experience. For myself, where I do not see such tumidity, I operate only in the faint hope of relieving a person, but I never do it with confidence; but where I see the tumidity, I treat with assurance. If one comes to you with a condition of neuralgia in the lower jaw, and if you find tumidity, and rheumatism as well, you may fairly think of the lesion as lying with the rheumatic condition, and endeavor to verify your diagnosis by antirheumatic treatment.

Neuralgia along the line of the temporal artery is always associated with the auriculo-temporal nerve. Its cause, if my experience be not unique, lies with exhaustion. It is a pain rare in men, but common to weak, over-worked store-girls. Indication is for rest and change. The sea-shore is the best place to send the patients. As a tonic, my individual experience favors the use of a formula as follows:

℞ Hydrarg. bichlor., gr. i;

Liq. arsenici chlor., ʒss;

Tinct. ferri chlor., ʒii;

Acid. hydrochlor. dilut., ʒi;

Syr. sarsaparillæ, ℥vii. M.

Dose.—Tablespoonful one-half hour after meals.

Section of the nerve, and removal of an inch, has never failed in my own hands to result in local relief.

Next, pain of neuralgic character located persistently about the region of the infraorbital foramen, like that of the mental locality is not unlikely to have as its cause a periosteal thickening of its membrane as it associates with the opening.

Section of the nerve in these cases is to be practised by removing the anterior wall of the antrum and making the division well back of the lesion. If the nerve be cut in front of the foramen, the result is transference of the expression of irritation to some other locality.

Next, pain about the bridge of the nose. This I am to infer out of my individual experience to associate with a lesion somewhere about the ophthalmic division of the trifacial. I think this pain is persistently defying. It is difficult, as a performance, to cut this first branch, which, as remembered, comes from the brain-case through the sphenoidal fissure, being in close relation with the optic nerve. I am satisfied that in every instance this neuralgia is expressive of a local lesion.

Next is the form of neuralgic pain that is unlocatable, being today in this bone or part, and to-morrow somewhere else. For myself, I am at a great loss here, save a lesion discovers itself plainly and openly. Cause may be constitutional or it may be local. An only thing to be done is to look, first, locally; second, systemically.

I had the case of a man whose suffering was so great that at night he would run around the streets of a village in which he lived, screaming with agony. I did seven operations for him; and one day he said, "Either cure or kill me." I said I did not know what to do for him save to take away his lower jaw. I took the bone away, and he has not had a twinge of pain since.

One of the most remarkable cases—a case you will see the obscurity of—was as follows: A gentleman came to me with a pain in the eye. He was another one who would shoot himself if he was not to be cured. I became much interested in him. He would come to my office, and, while describing his suffering, would suddenly jump up and crawl under the table, where he would scream from pain. I said to myself, "Effect and cause. Can I not, by great effort, by giving time, by spending myself on this man, discover the cause in which the effect exists, and by removing it cure him?" That man had searched for relief through the different cities of Europe. He had been in your city, where he had been under the care of some of your best practitioners. He had been in hospitals without number without receiving any relief, all of which naturally increased an interest as to finding out, if possible, what was the matter with him. I went all around the circle I was talking about a little while back. But, going all around it, I did not find anything to explain the pain. I began to use atropia in the eye. I made a strong solution of it, but it seemed to have no more effect than if it had been put on a brick-bat. However, I kept on for weeks, and, finally, I did get the iris contracted, and I saw what seemed to be a little tumor coming to the edge of it. I said, "This is the cause," and I kept up the action of the mydriatic until I discovered a cysticercus. I watched this with great interest, not being disposed to show it until I was positive. One day I found it boring into the crystalline lens. I cured that man by removing the parasite, and he has had no twinge of pain in the part since.

We never have a condition of disease without there is the "dis," while our ability to discover the "dis" lies, as before suggested, in the experience had. Some of us direct our experience profoundly in one direction; some in another. That is one great beauty about professional fellowship. I go to one gentleman, who has given a great deal of attention to one class of troubles. I ask him to give me his opinion on a certain case; then he comes to me in turn and asks my opinion on something else, just as we are doing to-night.

I will not detain you longer, because we are here to be of use to one another, and it comes now my turn to listen to what others have to tell in the way of experiences.

